# A resource to explore the discovery of rare diseases and their causative genes

**DOI:** 10.1038/s41597-021-00905-y

**Published:** 2021-05-04

**Authors:** Friederike Ehrhart, Egon L. Willighagen, Martina Kutmon, Max van Hoften, Leopold M. G. Curfs, Chris T. Evelo

**Affiliations:** 1 Department of Bioinformatics - BiGCaT, NUTRIM School of Nutrition and Translational Research in Metabolism, Maastricht University, Maastricht, The Netherlands; 2 Governor Kremers Centre - Rett Expertise Centre, Maastricht University Medical Center, Maastricht, The Netherlands; 3 Maastricht Centre for Systems Biology (MaCSBio), Maastricht University, Maastricht, The Netherlands

**Keywords:** Data integration, History, Literature mining

## Abstract

Here, we describe a dataset with information about monogenic, rare diseases with a known genetic background, supplemented with manually extracted provenance for the disease itself and the discovery of the underlying genetic cause. We assembled a collection of 4166 rare monogenic diseases and linked them to 3163 causative genes, annotated with OMIM and Ensembl identifiers and HGNC symbols. The PubMed identifiers of the scientific publications, which for the first time described the rare diseases, and the publications, which found the genes causing the diseases were added using information from OMIM, PubMed, Wikipedia, whonamedit.com, and Google Scholar. The data are available under CC0 license as spreadsheet and as RDF in a semantic model modified from DisGeNET, and was added to Wikidata. This dataset relies on publicly available data and publications with a PubMed identifier, but by our effort to make the data interoperable and linked, we can now analyse this data. Our analysis revealed the timeline of rare disease and causative gene discovery and links them to developments in methods.

## Abstract (summary)

**Table:** 

Measurement(s)	Gene_Associated_With_Disease • genetic disorder
Technology Type(s)	digital curation
Factor Type(s)	disease
Sample Characteristic - Organism	Homo sapiens

Machine-accessible metadata file describing the reported data: 10.6084/m9.figshare.14140661

## Background & Summary

Descriptions of unusual diseases date back until the ancients, but rare genetic diseases are a relatively new chapter in the history of medicine as genes as carriers of hereditary diseases were only discovered in the middle of the last century (see *e.g*. Reflections on medicine and art).

Identification of the disease-causing mutation in the plethora of genetic variation an individual human carries is a difficult task in the diagnosis of rare diseases. A typical human individual has about 4.1–5 Mio variants compared to the reference genome^1^. For the identification of the disease-causing variant, experts cross-check with variant databases and use variant pathogenicity prediction algorithms. There are several bioinformatics workflows available to go from the raw data to the detection of the causative mutation, see e.g. Gilisen *et al*.^2^. Within this process mapping of genetic data, identifiers and information is required in several ways.

Many genotype-phenotype databases link information about rare diseases, their causative genes, and gene variants, respectively^3^. Some well-known databases like OMIM (www.omim.org), Orphanet (http://www.orpha.net), and DisGeNET^4^ include provenance - *e.g*. in the form of manually curated or text mining derived literature lists. DisGeNET provides an extensive collection of linked data including a semantic model. Orphanet does as well but focuses more on patient care related information. OMIM is the online version of the genetic (Mendelian) disease encyclopaedia that provides information in the form of a literature list for a disease or a gene and provides gene-disease mapping spreadsheets, *e.g*. a morbid map^5^. The given literature lists, which support gene-disease associations, are useful to provide accumulated provenance for this association. However, none of these databases provides a list or a dataset that links the unique publication, which described the link between the gene and the disease first. This information is very useful for historic perspectives on genetic and rare disease research.

We produced a mapping dataset, which links rare, monogenic diseases to their causative genes (and *vice versa*), backed up by the publication which proves the genetic cause for a disease for the first time, or historic provenance. The data are annotated with OMIM identifiers for the disease, gene identifiers (HGNC^6^ and Ensembl^7^) for the gene, and PubMed identifiers (PMIDs) for the literature. In order to describe the content on this spreadsheet and to provide it in a structured way we developed a dedicated version of the DisGeNET semantic model to produce a resource description framework (RDF) file and a set of nanopublications. Nanopublications are short information sequences built from identifiers and ontologies with embedded provenance, that especially allow data mining and automated read-out^8^. Nanopublications are usually accompanied by a meta data header, and if correctly formed and made available they are automatically FAIR (Findable, Accessible, Interoperable and Re-usable^9^). There are not many tools yet that can process and use nanopublications, but due to their structure, they can be implemented in graph based databases (e.g. Wikidata, as was done in this study) and can be used in graph based applications.

We produced a linkset based on this data that was formatted to be used directly with the Cytoscape app CyTargetLinker^10^. Cytoscape is a popular network analysis software^11^. The linkset can be used for instance for genetic variants or gene expression analysis linking genetic variants to genes or pathways^12^.

Using the semantic web linksets provided for our dataset, different interesting facts can be retrieved, *e.g*. a timeline of discovery of genes responsible for rare diseases, the links between different diseases caused by the same gene or between a disease that is known to be caused by different genes independently. It can also be used as a starting point for more extensive analyses about the landscape of diseases present in the underlying databases.

## Methods

### Workflow

**Fig. 1:**
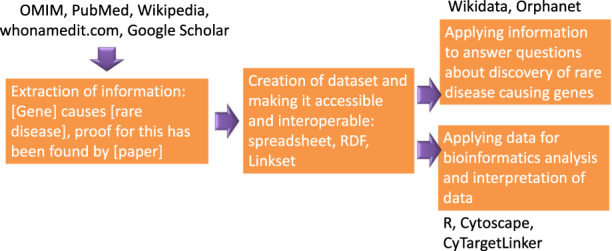
Workflow of information acquisition, creation of dataset and downstream analysis.

In short, we first collected, extracted, and reviewed the information to create the dataset. Second, the dataset was created and made accessible in three different ways: spreadsheet, RDF, and linkset (for CyTargetLinker). Third, the information from the dataset was used to retrieve information about the discovery of rare disease causing genes and, fourth, applied in different analysis use cases. The workflow is shown in Fig. 1.

### Creation of the dataset

The OMIM database was accessed to generate a list of all known gene-disease relationships that contained the OMIM ID and the disease labels for the diseases and the gene names. From this, rare monogenic diseases were manually identified and extracted. Wherever possible we compared with Orphanet to determine the abundance of a disease with a cut-off of 1:1000, which is less exclusive than the EU definition^13^. In some cases, we had to use our own judgement for diseases that are described as uncommon. The first description of the disease was manually retrieved by literature research using different resources like OMIM, PubMed, Whonamedit [https://www.whonamedit.com/], and Wikipedia. If no clear first description publication could be identified, the oldest publication found was used, which was typically the oldest reference in the literature list on OMIM. This step was also done by manual curation. Publications were annotated in the dataset with PMIDs. For the causative gene for the disease we replaced the gene name from OMIM with the stable gene identifier from Ensembl and the appropriate HGNC symbol using Ensembl BioMart mapping. Provenance for the first publication proving that a particular disease is caused by a specific gene was manually identified based on the literature provided on OMIM and other sources (PubMed, Wikipedia, Google Scholar). The PMID of this publication was added. Table 1 shows the structure of the final dataset.

**Table 1:** The spreadsheet data content and structure.

Gene information		Gene - disease association provenance	Disease information		
ENSEMBL Gene ID ENSID	HGNC symbol	PMID Gene-disease association	Disease OMIM ID	Disease name	PMID Disease
ENSG00000188536	HBA2	1115799	604131	Thalassemia, alpha-	1115799

The dataset (version 2) contains 4565 gene-disease associations with provenance, of which 4292 are unique gene-disease associations. The difference is due to multiple PubMed identifiers for provenance of some diseases, and in a few cases, it was not possible to identify which publication was published first. There are 3154 unique genes (according to their Ensembl identifiers) and 4166 unique diseases (based on OMIM identifiers) in the dataset.

### Creation of RDF/nanopublications

To create an RDF file (according to standards as stated here: https://www.w3.org/TR/hcls-dataset/) a modified version of the DisGeNET RDF model was developed. The code to create the RDF is available at: https://github.com/BiGCAT-UM/raredisease-omim/tree/master/rdf. All data files are available in this Figshare collection, and the actual version discussed here is version 3^14^.

Nanopublications were generated from spreadsheets in tab delimited (TSV) format available from Figshare using a custom Groovy script that makes use of nanopub-java^15^, available at https://github.com/BiGCAT-UM/raredisease-omim/tree/master/nanopub. The nanopublications make use of the Semantic science Integrated Ontology (SIO)^16^, identifiers.org^17^, VoID^18^, Dublin Core^19^, and the NCI Thesaurus^20^.

### Creation of linkset for CyTargetLinker

CyTargetLinker is a Cytoscape app, which facilitates network extension using information given in linksets. These linksets are usually derived from external databases and contain *e.g*. gene-microRNA or drug-drug target relationships. The gene-rare disease linkset for CyTargetLinker was created as described in Kutmon *et al*.^10,21^. It includes the PMIDs for the first description as the annotation of the edges. We used a Java program (available here https://github.com/CyTargetLinker/linksetCreator) to convert tab delimited text files into an XGMML formatted linkset, which is also available from the CyTargetLinker linkset repository (https://cytargetlinker.github.io/pages/linksets).

## Data Records

The tab separated spreadsheet data (TSV) collection of the gene rare disease provenance data (gene-RD-provenance_v2.1.txt) is available in this Figshare collection^14^. The application specific versions of the gene-RD-provenance dataset are available on these resources: the linkset for CyTargetLinker in the CyTargetLinker repository under disease related linksets, the current version is 2, the nanopublications in RDF format are also available from the above mentioned Figshare collection. To support the use of the data on Wikidata^22^ we checked the availability of all rare diseases, genes and publications there. All genes were already available and we therefore could easily add the publications using the PMIDs. Provenance of the first description of the disease was added to the disease statement in Wikidata. All diseases that had an associated PMID were also included; the others will gradually be added over time. Provenance of the first description of the causative gene was added to the genetic association statement of the disease. About 800 publications were added to Wikidata. All additions to Wikidata mentioned here were uploaded using QuickStatements (https://tools.wmflabs.org/quickstatements/#/).

For importing in R we recommend to disable quoting and enabling headers (read.table(file = “Gene-RD-Provenance_V2.1.txt”, quote = “”, sep = ‘\t’, header = TRUE).

## Technical Validation

### Quality of the data

The original data in tabular format can be uploaded as a Google Spreadsheet, Google provides an API to access the data, as used below. The data uses common database identifiers provided by the underlying resources to identify biological entities, such as the Ensembl gene identifier, HGNC symbol, PMID, and OMIM identifier of the disease. In the RDF we support open standards for these identifiers using identifiers.org, URIs that are unique, persistent and resolvable. The technical quality of the data further follows from the ability for error free conversion into nanopublications.

### Validity of the nanopublications

The RDF nanopublication format was selected as one of the formats to share the data, a format with a clear open standard and the ability to use ontologies. The generation of the nanopublications catches many technical errors in the original data, such as incorrect identifiers, which result in incorrectly formatted RDF content. The used nanopub-java library performs further validation of the correctness of the nanopublications. The resulting TRIG file with nanopublications was technically validated with the *rapper* tool (http://librdf.org/). The RDF uses various ontologies, ensuring that data can be expressed using common terminology and making it interoperable. The data has been deposited in the nanopublications network^23^.

## Usage Notes

### Bibliographic information

As an example, we looked up the publication dates and other relevant information for the two different lists of PMIDs, extracted from the TSV file. To do this, we used these PMIDs to query for the first descriptions of rare disease and the first descriptions of gene-disease relationships in Wikidata. The SPARQL queries can be found here: https://github.com/BiGCAT-UM/pubmedWikidata.

#### Timeline of first descriptions of rare diseases

**Fig. 2:**
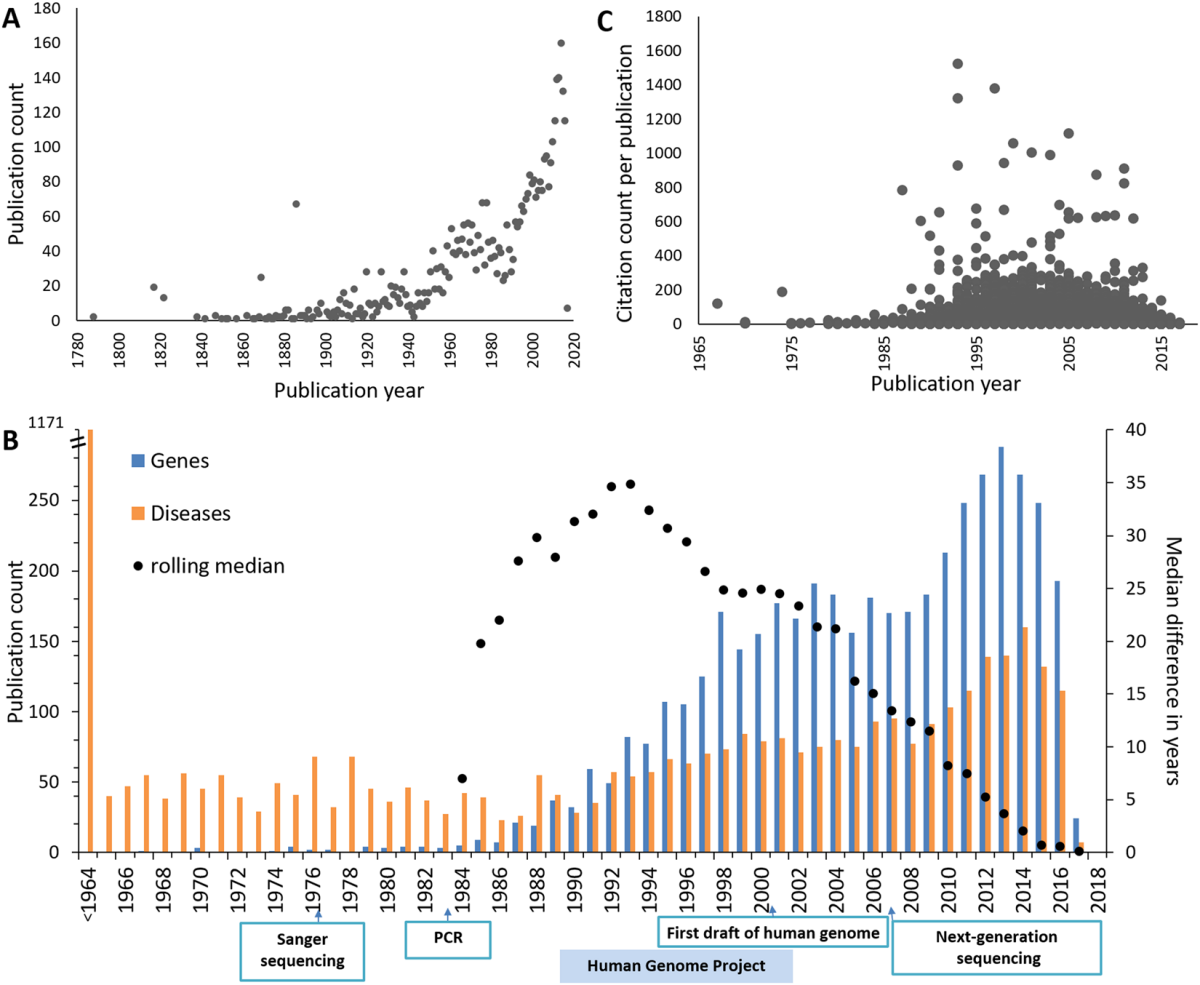
(**A**) Number of first descriptions of rare diseases per year. (**B**) Number of publications identifying new disease description (orange bars) and new gene-disease links per year (blue bars). The black dots indicate the (rolling) median number of years the diseases had been known before the causative gene was identified in that year. The data are displayed from the year 1984, from which there were constantly more than four genes per year discovered. (**C**) Total current citation counts for the papers shown for gene-disease relationship papers shown as blue bars in panel B shown for the year these were published. One dot represents one publication from our dataset.

To illustrate the use of our dataset we used the results of the SPARQL queries to find that the first description of a rare disease was from 1788 (Olof Ekman) about osteogenesis imperfecta (Fig. 2A). Later in 1988 and 1989, mutations in two collagen genes, *COL1A1*^24^ and *COL1A2*^25^, were identified as responsible for this disease. Several first descriptions of related phenotypes were later classified as separate diseases and were associated with different genetic causes. In 1817 James Parkinson wrote “*An Essay on the Shaking Palsy”* describing the disease which was later named after himself. By now, there are 19 different entries in OMIM named Parkinson’s disease (or a variety of) with different genetic causes (not to be mixed up with Wolf-Parkinson syndrome, which was named after Sir John Parkinson). A remarkable peak in discoveries occurred in 1886, when Charcot-Marie-Tooth disease was described, which was later sub classified in about 58 different subtypes and responsible genes. Additionally, in this year first descriptions were made about Pheochromocytoma and Multiple endocrine neoplasia (each with about 5 different subtypes and responsible genes). After 1901 there was no year passing without at least one new description of a rare disease.

#### Timeline of rare disease-gene relationship discovery

Using the PMIDs of the publications stating for the first time that a certain disease is caused by a specific gene, Wikidata was queried with the same query as described above but this time with the PMIDs of the gene-disease association provenance. In Fig. 2B we plotted the number of publications about gene-disease relationships that appeared per year. In this dataset, the earliest gene-disease link is from 1967 when Seegmiller *et al*.^26^ found that a neurological disorder (which was described first three years earlier and only later the name Lesch-Nyhan Syndrome was established^27^) was caused by the absence of hypoxanthine-guanine phosphoribosyltransferase. The discovery rate increased following the invention of both Sanger sequencing and PCR, reached a plateau between about 1996 and 2006 and increased again after development of next generation sequencing. The decline of the discovery rates after 2013 can easily be explained because at that time causative genes for all known genetic diseases had already been found and new causal relationships could only be identified for newly found diseases.

In parallel, the time difference since a disease was described first until the causative gene was identified was at maximum in the early 90s with 37 years and declined since then to almost zero after 2014 (Fig. 2B, rolling median). The data before 1990 is very scattered as the discovery of genes was rare and had just started. After establishing next generation sequencing in 2007, by about 2013 it became a common standard that together with the description of a new genetic disease the genetic cause is also identified and published in the same document. The time span does not drop to zero because new rare genetic causes of long known diseases (e.g. Charcot-Marie-Tooth disease) are still occasionally discovered. This is reflected in the data; the mean and the median drift apart after about 2004 indicating increasing scattering of the data.

#### In which journals are newly discovered diseases or genes published

Here, we demonstrate how to query in which journals newly discovered diseases or new causative genes for rare diseases are preferably published. First descriptions of rare diseases had a PMID in 3144 cases; for new genes causing a rare disease 4263 of in total 4565. To get the journals in which these papers have been published, the SPARQL endpoint of Wikidata was accessed using the queries mentioned above. Table 2 lists the top 10 journals in which new diseases and new causative genes have been described. According to the data, the most important journals for publishing both, new rare diseases and newly identified genes for rare diseases, are the American Journal of Human Genetics (14.9% for diseases and 26.7% for genes) and Nature Genetics (4.7% for diseases and 18.4% for genes). In total, for new rare diseases we identified 364 different journals, and 197 journals for genes. This may be due to the broader spectrum of medical disciplines, and therefore journals in which the first observation of a now rare disease was described, as well as the broader timeframe in which these were published.

**Table 2:** Journals, in which the new discovered diseases or genes causing a rare disease were published.

journal	count	%
**First description of a new disease (100% = 3144)**		
American Journal of Human Genetics	457	14.9
Nature Genetics	145	4.7
American Journal of Medical Genetics Part A	137	4.5
Journal of Medical Genetics	136	4.4
Human Molecular Genetics	122	4.0
The New England Journal of Medicine	121	3.9
Journal of Clinical Investigation	78	2.5
Neurology	77	2.5
The Journal of Pediatrics	68	2.2
Proceedings of the National Academy of Sciences of the United States of America	59	1.9
**First description of new genes causing a rare disease (100% = 4263)**		
American Journal of Human Genetics	1137	26.7
Nature Genetics	786	18.4
Human Molecular Genetics	266	6.2
Journal of Medical Genetics	175	4.1
The New England Journal of Medicine	148	3.5
Journal of Clinical Investigation	136	3.2
Proceedings of the National Academy of Sciences of the United States of America	129	3
Science	121	2.8
Nature	99	2.3
Human Mutation	94	2.2

#### Citation counts

The citation count of the publications was retrieved indirectly by querying Wikidata. Next to direct entries, citation information in Wikidata is supplemented with information from PubMed and CrossRef via the WikiCite project^28^, which at this moment covers about 59% of publishers (Open Citations, https://i4oc.org/, status Sep 2019) and Wikidata has about 12.5% of all citations (status Aug 2018).

Figure 2C shows the citation count distribution across the publication year for the first description of a causative gene for a rare disease. The mean citation count for such a paper is 56. Among the top 10 of most cited papers are several, which identified rare, genetic causes for Alzheimer’s and Parkinson’s disease, Huntington’s disease, macular degeneration, Rett syndrome, Crohn’s disease, amyotrophic lateral sclerosis, and chromosome 9p-linked frontotemporal dementia. High citation numbers indicate that there is a lot of research done for these diseases. Low citation numbers or none at all may be due to the newness of the finding (less than 5 years), disagreement among researchers, non-reproducibility of the result or no “interest” in terms of grants and research capacity investment. Among the true “neglected” diseases, diseases for which the genetic cause has been identified before 2013 but had been cited only once by now are e.g. 3-ketothiolase deficiency, glucose 6-phosphate isomerase deficiency, Leber congenital amaurosis, sarcosinemia or nonsyndromic oculocutaneous albinism. Additionally, there are many disorders, which do not have a name and are described by their phenotype appearance (e.g. “Manifestations of X-linked congenital stationary night blindness in three daughters of an affected male: demonstration of homozygosity”). Apart from these, about 1000 gene-rare disease discoveries have not been cited yet (according to the available data).

The most cited first disease descriptions are about Inflammatory bowel disease 25, early onset, autosomal recessive (4080 citations), D-2-hydroxyglutaric aciduria 2 (1598 citations), Macular degeneration, age-related, 4 (1498 citations), and Osteogenesis imperfecta, type XII (1031 citations).

#### Authors

2641 (co)authors of papers, which first described a causative gene for a rare disease have an ORCID, which is about 15% of all these authors (https://github.com/BiGCAT-UM/literatureSummary). Table 3 gives the top ten authors with the most causal gene discoveries. 993 first rare disease description paper (co)authors (19.7%) have an ORCID. The lower number may in part be due to the fact that the description of rare disease ranges further to the past in which ORCID was not available.

**Table 3:** Top ten of authors with most gene discoveries in this dataset.

Author name	count
Arnold Munnich	58
Gudrun Nürnberg	44
Peter Nürnberg	38
Thomas Meitinger	35
Nicholas Katsanis	32
Friedhelm Hildebrandt	31
Jean-Laurent Casanova	31
Alexis Brice	30
Edgar A Otto	19
Bruno Dallapiccola	28

### Application of the dataset for analysis

#### Network of genes causing diseases

**Fig. 3:**
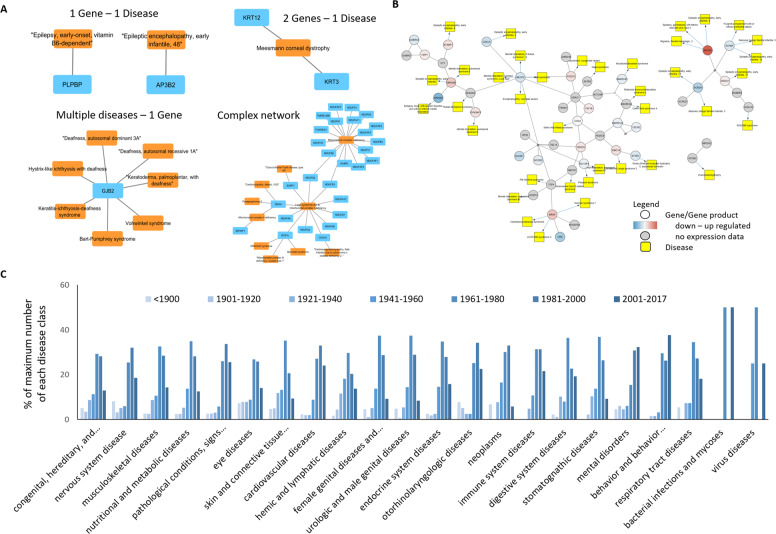
(**A**) Network of gene-rare disease relationships. Blue nodes are genes (HGNC symbols), orange nodes are diseases (OMIM disease names). (**B**) The Rett syndrome causing genes pathway from WikiPathways, https://www.wikipathways.org/instance/WP4312 was imported as a network to Cytoscape environment using the WikiPathways app of Cytoscape. Using CyTargetLinker app, the MECP2 network was extended to predict and visualize overlap of pathway genes causing other rare diseases provided by the gene-RD-Provenance_V2 linkset. The expression data was taken from Miller *et al*.^29^ and the data was originally produced by Lin *et al*.^30^. (**C**) Timeline of rare disease superclass descriptions in blocks of 20 years. The numbers are normalized to percentages of the maximum number of diseases in each disease superclass discovered (Table 4).

To investigate the dataset, a network of gene-disease relationships was created using Cytoscape software. The network analysis shows 2357 gene-disease causative relationships in which one gene causes one disease (Fig. 3A upper left). Provenance can be by one or more publications depicted in one or more edges connecting the nodes. 446 triplets of two genes causing one disease, or one gene causing two diseases, are found, whereas in the majority of these triplets there is one gene causing two diseases. This may be explained from the fact that disease phenotypes can be different, often leading to the description of two varieties of one disease, which were given separate identifiers in OMIM. 1226 genes and diseases are linked in more complex patterns (Fig. 3A bottom). Again, in the majority of these cases, there is one gene responsible for multiple diseases or varieties of disease. The largest complex contains mainly mitochondrial disorders and their associated diseases. Here, a lot of overlap between the diseases and causative genes is observed, possibly because several genes are contributing to the functionality of one complex.

#### Integration in network analysis

Based on this dataset we created a linkset for the Cytoscape plugin CyTargetLinker (see here how to use and create these linksets https://github.com/CyTargetLinker/linksetCreator). In Fig. 3B, the result of such a network extension is shown. The basic network is a rare disease pathway imported to Cytoscape from WikiPathways [https://www.wikipathways.org/instance/WP4312]. The expression data shown in the network was taken from Miller *et al*.^29^, and the data was originally produced by Lin *et al*.^30^. The network was then extended using the Gene-RD-Provenance_V2 linkset (available here: https://cytargetlinker.github.io/pages/linksets) to show which genes in the network may cause other rare genetic diseases. Examples for the identified diseases are SESAME syndrome, Glass syndrome, Mental retardation X-linked, etc. This linkset can in general be used to explore associated disease phenotypes for mis-regulated genes or deleterious genetic variants in networks.

### Linking data and information with other datasets, mappings and RDF based databases

DisGeNET provides mapping datasets (available at https://www.disgenet.org/downloads - Mappings) which allow mapping of OMIM identifiers to Concept Unique Identifiers (CUI) and Orphanet identifiers (ORPHA). Using our list of rare disease OMIM identifiers, it was possible to map 99.2% of them to CUI and 58.0% to ORPHA. With these mappings, it is possible to retrieve and integrate the specific information from different resources.

It is worth noting that the definitions of a disease vary between the different databases and thesaurus systems so there is a discrepancy in reported data output. E.g. OMIM disease identifiers can map to multiple ORPHA and *vice versa*.

#### DisGeNET RDF and database - identification of disease superclasses

Querying the DisGeNET database and RDF we found that 58.7% of the rare diseases are annotated with a disease superclass term from MeSH (medical subject headings). The top ten of superclass terms are listed in Table 4. The majority of rare diseases are annotated with “congenital, hereditary, and neonatal diseases and abnormalities”, “nervous system disease”, and “musculoskeletal diseases”. The dominance of nervous system diseases as consequences of genetic mutations was documented before with data from OMIM^31^.

**Table 4:** Top 10 MeSH superclasses for the rare diseases.

Top 10 MeSH terms	Count
congenital, hereditary, and neonatal diseases and abnormalities	1705
nervous system disease	945
musculoskeletal diseases	595
nutritional and metabolic diseases	549
pathological conditions, signs and symptoms	415
eye diseases	328
skin and connective tissue diseases	296
cardiovascular diseases	203
hemic and lymphatic diseases	182
female genital diseases and pregnancy complications	174

#### What classes of disorders have been discovered when

Linking publishing date of a rare disease description with MeSH disease superclass information from DisGeNET reveals trends in which class of diseases have been identified. Figure 3C shows a timeline in blocks of 20 years, showing for each block how many rare diseases belonging to the respective superclass have been described.

For most disease superclasses the discovery rate increases slowly, and they see the majority of discovery between 1961–2000. Some disease superclasses were discovered relatively early or late, probably due to the availability of diagnostic methods. Metabolic and endocrinological diseases for instance have their peak in 1961–1980 while mental disorders peak in 2001–2017. The latter might in part be due to increasing differentiation and definition of neuronal, mental and behavioural disorders.

## Data Availability

The code to create the RDF from a Google spreadsheet that can be created from the TSV file is available at: https://github.com/BiGCAT-UM/raredisease-omim/. The code to create the Linkset for CyTargetLinker is available at: https://github.com/CyTargetLinker/linksetCreator. The queries to retrieve information about the publications using the PMIDs in Wikidata can be found here: https://github.com/BiGCAT-UM/pubmedWikidata.
